# Mind-Body Exercise (Wuqinxi) for Patients with Chronic Obstructive Pulmonary Disease: A Systematic Review and Meta-Analysis of Randomized Controlled Trials

**DOI:** 10.3390/ijerph16010072

**Published:** 2018-12-28

**Authors:** Ke Wang, Shijie Liu, Zhaowei Kong, Yanjie Zhang, Jing Liu

**Affiliations:** 1 Department of Physical Education, Northwestern Polytechnical University, Xi’an 710072, China; wangke123@nwpu.edu.cn; 2 Department of Physical Education, Wuhan University of Technology, Wuhan 430070, China; liushijie0411@whut.edu.cn; 3 Faculty of Education, University of Macau, Macau, China; zwkong@umac.mo; 4 Health and Exercise Science Laboratory, Institute of Sports Science, Seoul National University, Seoul 08826, Korea; 5 Physical Education Unit, School of Humanities and Social Science, The Chinese University of Hong Kong, Shenzhen 518172, China; 6 Department of Martial Arts, Shanghai University of Sport, Shanghai 200438, China

**Keywords:** Mind-body exercise, mindfulness-based exercise, meditative movement, Wuqinxi, COPD, Randomized controlled trial

## Abstract

*Objective*: This study is the first meta-analysis investigating the rehabilitative effects of Wuqinxi for patients with chronic obstructive pulmonary disease (COPD). *Methods*: Five electronic databases (PubMed, Web of Science, Scopus, CNKI, and Wanfang) from inception until early November 2018 were searched. All randomized controlled trials (RCT) using Wuqinxi as the main intervention component were included for meta-analysis. The pooled effect sizes (Standardized mean difference, SMD) were calculated to determine the magnitude of the Wuqinxi intervention effect. Moderator analysis was only conducted for total training time. *Results*: Overall results of the meta-analysis indicated that Wuqinxi exercise significantly improved exercise capability (*SMD* = 1.18, 95% CI 0.53 to 1.84, *e* < 0.001, *I*^2^ = 84.97%), FEV1 (*SMD* = 0.44, 95% CI 0.12 to 0.77, *e* < 0.001, *I*^2^ = 33.77%), FEV1% (*SMD* = 0.59, 95% CI 0.24 to 0.93, *e* < 0.001, *I*^2^ = 63.79%), FEV1/FVC (*SMD* = 0.65, 95% CI 0.37 to 0.93, *e* = 0.006, *I*^2^ = 44.32%) and CCQ (*SMD* = 1.23, 95% CI 0.31 to 2.14, *e* = 0.01, *I*^2^ = 93.32%). *Conclusions*: With no occurrence of adverse event, clinicians could try to incorporate Wuqinxi exercise into their first-line rehabilitation regime for COPD patients.

## 1. Introduction

Currently, environmental problems, especially air pollution, are affecting respiratory health on a global scale [1]. Of note, chronic obstructive pulmonary disease (COPD) is among the leading kinds of chronic respiratory diseases that are among the leading causes of mortality and morbidity all over the world [2]. COPD is characterized by progressive, incurable airflow limitation, and is commonly caused by exposure to noxious particles or gases that result in airway or alveolar abnormalities [3]. Patients with COPD have a significant number of coexisting problems such as physical inactivity, psychological issues (anxiety and depression), cardiovascular disease and lung cancer [4]. The Global Burden of Disease study reported that the prevalence of COPD increased by about 45% compared to 1995, and more than 170 million individuals were suffering from COPD in 2015 [5]. Given the rapid increase of the incidence of COPD, the World Health Organization (WHO) predicts that the morbidity and the number of deaths from COPD will continue to increase in the next decade, becoming the third leading causes of death worldwide [6].

Given the severe morbidity and death situation from COPD, a great deal of effort and research has gone into understanding how physical activity may combat COPD through enhancing exercise capacity and health-related quality of life, and reducing symptoms [7]. For example, a randomized controlled trial about physical activity in individuals with COPD has been published, showing that enhancing physical activity can effectively increase the forced vital capacity and quality of life [8]. The extant meta-analysis paper indicated that daily physical activity may have significant association with mortality, exacerbations and whole-body health to patients with COPD [9]. Recently, a new exercise strategy is emerging for overcoming COPD. Wuqinxi, as one of traditional Chinese mind-body exercises (Tai Chi and Baduanin Qigong) [10,11,12], is an important part of traditional rehabilitation therapy in ancient and modern China [13]. It is characterized by concentration, relaxation, mediation, regular breathing, and gentle body movement. As compared to traditional chinses Tai Chi, Wuqinxi is considered an easy-to-learn Qigong exercise, including only five forms that can be practiced individually in different places (e.g., home, office) [14]. It is also known as the “five animals exercise”, which was created by well-known Chinese physician Huatuo in the Donghan Dynasty [14]. Trainees of Wuqinxi imitate the specified technical action and breathing of five animals (tigers, deer, bears, apes and birds), and pay attention to integrating the regulation of body, respiration, and mind into “one” [15]. Growing evidence exists that Wuqinxi, as a low- to moderate- intensity aerobic exercise, can positively contribute to human health across different ages, especially concerning conditions such as primary osteoporosis [16], lumbosacral multifidus in the elderly [17], and physical health in the young [18].

Moreover, for the recent decade, a number of original studies were performed to examine the effects of Wuqinxi on the physical and psychological capacities for COPD patients during data retrieval as the Wuqinxi is simple and easy to learn [19,20,21,22,23,24,25,26]. In particular, Wuqinxi is well accepted and popular in China today, and os even likely to become a popular mind-body exercise all over the world, i.e., in Singapore [27], Korea [28] and Germany [29]. However, the absence of systematic review led the authors to critically appraise and synthesize results, and to fill in the gap about people’s understanding of the evidence-based clinical practice of Wuqinxi. It was really necessary that researchers conduct a systematic review and meta-analysis to critically evaluate the efficacy of practicing Wuqinxi among COPD patients, and the benefits of this promising exercise can be known by more people in the world.

## 2. Materials and Methods

This review was conducted in accordance with the Preferred Reporting Items for Systematic Review and Meta-Analysis (PRISMA) guidelines [30].

### 2.1. Data Sources

Five electronic databases (PubMed, Web of Science, Scopus, CNKI, and Wanfang) from inception until early November 2018 were searched based on two groups of keywords in a combined manner: (1) COPD or chronic obstructive pulmonary disease; (2) Wuqinxi, five-animal boxing, five animals, five animals exercise, five animals play, or traditional health Qigong; (3) Randomized controlled trial, or controlled study. Investigators also cross-checked the reference lists of those relevant studies identified at the initial stage. The literature search was performed by one of the investigators of the present study.

### 2.2. Inclusion Criteria and Study Selection

The included studies met the criteria if they: (1) were published in a peer-reviewed journal (2) were a randomized controlled trial, RCT; (3) recruited human subjects diagnosed with chronic obstructive pulmonary disease (COPD); (4) used Wuqinxi as the main intervention component, while the control condition involved other treatment or no intervention (e.g., wait-list or unaltered lifestyle); (5) reported at least one health-associated outcome. Any studies that did not meet the aforementioned inclusion criteria were excluded from this review. One of the investigators (S.L.) first screened all retrieved documents based on their abstracts and titles, followed by full-text assessment of the remaining articles against the predetermined inclusion criteria. To minimize bias of study selection, another investigator (J.L.) of this review independently performed the initial screening and assessment. Discussion with the third author was required to reach a consensus if a discrepancy on study selection occurred between the two investigators.

### 2.3. Assessment of Study Quality

Assessment study quality of included studies used the Physical Therapy Evidence Database (PEDro) scale [31,32,33], which includes 11 items: eligibility criteria, randomization, allocation concealment, baseline equivalence, blinding of stakeholders (participants, instructors, and assessors), retention rate of ≥85%, intention-to-treat analysis (missing data management), between-group comparison, and point measure and measure of variability. Given that only individuals diagnosed with COPD were included, there was no need to include “eligibility criteria” in the present review. Furthermore, to optimize the health benefits of COPD patients, it was decided that Wuqinxi exercise should be integrated with other treatments (drug therapy or usual care), but such combinations can affect the integration of study results. Under this condition, we added an item to objectively assess whether Wuqinxi was isolated or not [34,35,36]. Finally, a maximum of 9 points can be obtained for each individual study.

### 2.4. Data Extraction and Synthesis

Two investigators (S.L. and J.L.) independently extracted data using a standardized form which included the following information: reference (author and year of publication), location and language of publication, characteristics of patients (sample size and attrition rate, mean age/age range, and course of disease), intervention protocol (weekly training dosage and duration), measuring outcomes (exercise capacity, Lung function and quality of life) and measurement, and adverse event and follow-up assessment. A Meta-Analysis Software (Bio. Stat. Inc., Englewood, NJ, USA) was applied to synthesize the quantitative data (mean and standard deviation of each group at baseline and post-intervention and sample size of each group [26]. Pooled effect size (standardized mean difference, SMD) can be obtained; (1) small effect size = 0.2; (2) medium effect size = 0.5; (3) large effect size = 0.8. Between-study heterogeneity was assessed using the *I*^2^ statistic (small = 25%, moderate = 50%, and large = 75%) [37]. The results were calculated using a random-effects model with a 95% confidence interval (CI).

## 3. Results

### 3.1. Study Selection

**Figure 1:**
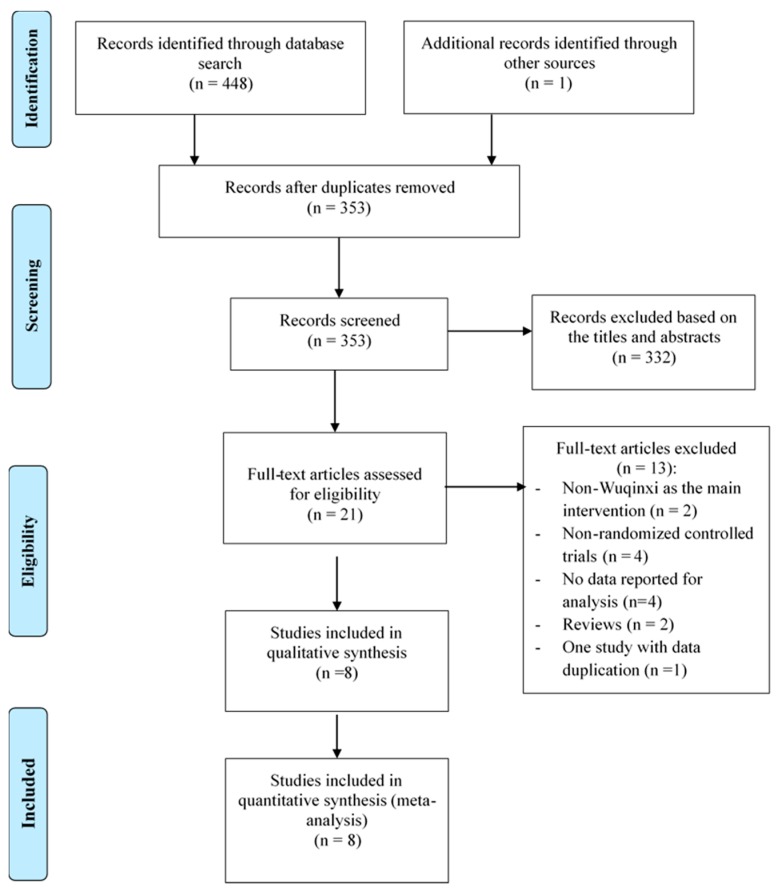
Flow of study selection.

In total, 449 records were retrieved, all of which were published in peer-reviewed Chinese journals. Such a low number of studies may be attributed to the fact that Wuqinxi Qigong is a new research topic, which only attracts Chinese scholars at the current stage. After examining for duplicates (*n* = 96), 332 irrelevant articles were removed based on title and abstract screening. The remaining of 21 potentially relevant articles were further assessed against the inclusion criteria, and 13 were excluded (non-Wuqinxi as the main intervention = 2, non-randomized controlled trials = 4, no data reported for analysis = 4, reviews = 2, and one study with two publications = 1). The study selection is presented in Figure 1.

### 3.2. Study Characteristics and Methodological Quality

The characteristics of the 8 selected randomized controlled trials are presented in Table 1 and Table S1. All peer-reviewed articles were published in Chinese-language journals. In total, 687 COPD patients (Wuqinxi intervention = 317 and control groups = 380) were included in this review, with an attrition rate ranging from 2.7% to 10.34%. These COPD patients ranged between 55.12 and 74.24 years of age on average, with the mean duration on their course of disease of 6.25 to 14.78 years. Wuqinxi in four studies was integrated with either drug therapy [20,21,23] or typical care [26]. In the control conditions researchers used either an isolated model (usual care, drug therapy, or Tai Chi) or a combined model such as breathing techniques plus drug therapy or walking training plus usual care. The intervention duration in Wuqinxi varied from 12 weeks to 24 weeks; the longest follow-up period was six months. Weekly training sessions occurred between 3 and 14 times (morning and afternoon session per day), with each session lasting 30 to 45 minutes.

Table 2 depicts the methodology quality of the trials, included building on the revised PEDro scale. Total scores across selected studies ranged from 6 to 8. Points were deducted due to studies lacking allocation concealment [20,22,24,25], blinding of assessors [19,20,22,23,24,25,26], and isolated Wuqinxi intervention [20,21,23,34]. Only one study reported the blinding of the assessor. Half of the eligible studies presented concealed allocation and used the combination of Wuqinxi and drug therapy/usual care.

### 3.3. Meta-Analysis of Outcomes Measured

**Figure 2:**
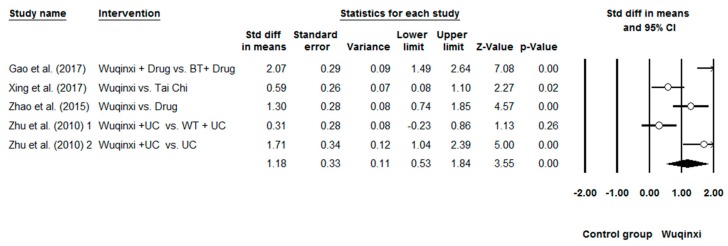
Effects of Wuqinxi on 6-minute Walking Test (BT = breathing training, WT = walking training, and UC = usual care).

**Figure 3:**
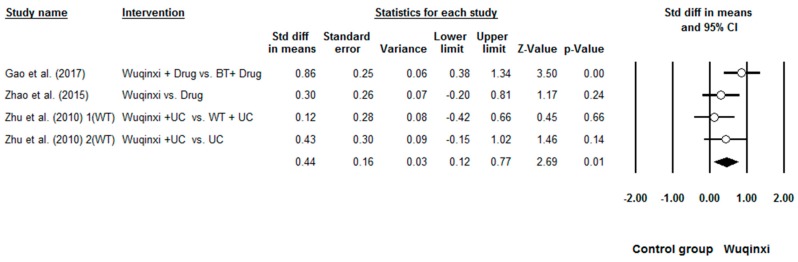
Effects of Wuqinxi on FEV1 (BE = breathing training, WT = walking training, and UC = usual care).

**Figure 4:**
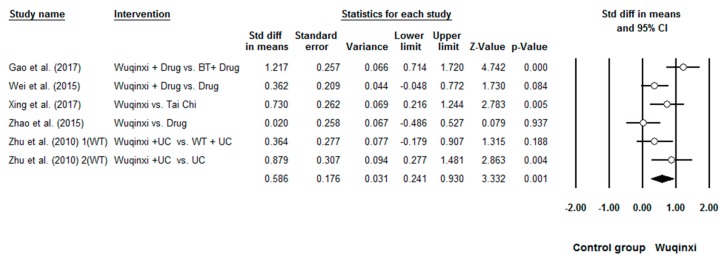
Effects of Wuqinxi on FEV1% (BT = breathing training, WT = walking training, and UC = usual care).

**Figure 5:**
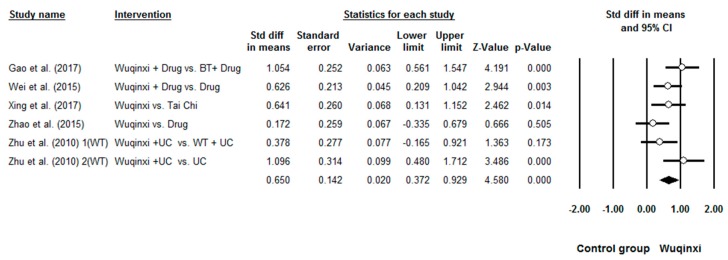
Effects of Wuqinxi on FEV1/FVC (BT = breathing training, WT = walking training, and UC = usual care).

**Figure 6:**
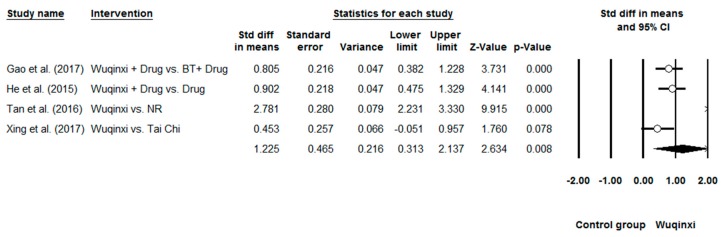
Effects of Wuqinxi on quality of life as measured by CCQ (BT = breathing training, NR = not reported).

Researchers investigated the effects of Wuqinxi on five health outcomes (6-MWT, FEV1, FEV1%, FEV1/FVC, and CCQ) of COPD patients. Study results are in favor of the positive effects of Wuqinxi on 6-MWT (*n* = 5), FEV1 (*n* = 4), FEV1% (*n* = 6), FEV1FVC (*n* = 6) and CCQ (*n* = 3) of COPD patients. Furthermore, the overall results of the meta-analysis indicated that Wuqinxi Qigong exercise significantly improved the 6-MWT (*SMD* = 1.18, 95% CI 0.53 to 1.84, *e* < 0.001, *I*^2^ = 84.97%; Figure 2), FEV1 (*SMD* = 0.44, 95% CI 0.12 to 0.77, *e <* 0.001, *I*^2^ = 33.77%; Figure 3), FEV1% (*SMD* = 0.59, 95% CI 0.24 to 0.93, *e <* 0.001, *I*^2^ = 63.79%; Figure 4), FEV1/FVC (*SMD* = 0.65, 95% CI 0.37 to 0.93, *e* = 0.006, *I*^2^ = 44.32%; Figure 5) and CCQ (*SMD* = 1.23, 95% CI 0.31 to 2.14, *p* = 0.01, *I*^2^ = 93.32%; Figure 6) in Table 3.

### 3.4. Moderator Analysis

Meta-regression was performed to determine whether the potential continuous moderator (total training time) influenced the outcomes. Study results indicated that longer Wuqinxi intervention was significantly associated with better performance; 6MW (*β* = 0.00014, *Q* = 10.33, df = 1, *e* = 0.001), FEV1% (*β* = 0.00011, *Q* = 9.04, df = 1, *e* = 0.003), FEV1/FVC (*β* = 0.00008, *Q* = 4.38, df = 1, *e* = 0.036), and CCQ (*β* = 0.00007, *Q* = 4.18, df = 1, *e* = 0.04). However, no significant relationship between total training time were observed on FEV1 (*β* = 0.00007, *Q* = 3.50, df = 1, *e* = 0.06). Results of the meta-regression are presented in Table 4. 

## 4. Discussion

Our study is the first meta-analysis to objectively synthesize the existing literature regarding the beneficial effects of Wuqinxi for COPD patients. Study results of the present review indicated that Wuqinxi has the potential to improve exercise capability and lung function of COPD patients. Furthermore, this traditional Chinese mind-body exercise could be integrated with first-line treatment like typical care or drug therapy to optimize the health outcomes of COPD patients. In addition, the results of our meta-analysis are consistent with previous reviews that investigated the rehabilitative effects of other mind-body exercises (Tai Chi, Yoga, Baduanjin) for COPD patients [38,39,40,41]. Detailed information about the results of the present review is also presented below. 

Exercise capacity deterioration in patients with COPD has been well-established [42]. Currently, no pharmacological treatment could hinder the progress of exercise capability of COPD patients, but exercise training has been shown to have positive effects on reducing disability [43]. Given that COPD patients exhibit very low exercise tolerance, Wuqinxi, as a traditional Chinese mind-body exercise, is characterized by slow bodily movement, limb-stretching, psychosomatic relaxation, breathing control, and meditative stage of mind, seems to reasonably suitable. No adverse event occurred during the Wuqinxi intervention period, which supports our belief that that this type of exercise modality is a safe rehabilitation program for COPD patients. The overall results of the meta-analysis indicated that Wuqinxi has a large effect on the exercise capabilities of COPD patients. Meanwhile, a significant heterogeneity may have been present in the Gao et al. study [21]. They demonstrated larger effects on all outcomes (including exercise capability) when compared with effect size of other individual studies. Furthermore, large effects of Wuqinxi were found on all outcomes. To observe the training protocol in this study, we found that COPD patients participated in both morning and afternoon sessions each day, with each session lasting only 30 minutes. This implied that this training mode (frequency and intensity) seems to be an optimal exercise prescription for COPD patients.

Lung function decline in COPD patients, assessed as expiratory airflow reduction (FEV1, FEV1%, and FEV1/FEV1%), ranged from 35 to 79 mL/year [44]. Results of our meta-analysis indicated moderate effects of Wuqinxi on lung function, with individual pooled effect sizes of 0.44 to 0.75. Although positive results were observed in terms of the pooled effect size, significant levels were not reached in some aspects such as FEV1 [25,26], FEV1% [23,25,26], and FEV1/FVC [25,26]. Such non-significant results for these outcomes may be partially explained by use of active controls. For instance, in the study of Zhu et al. [26], a control group was given walking exercise and typical care to treat COPD; it is likely that a synergistic effect of walking exercises/usual care occurred, leading to a comparable effect to the Wuqinxi-usual care. However, there was a trend that patients in Wuqinxi group had a greater improvement in lung function compared with the walking group, implying that patients who undertook the Wuqinxi exercise may obtain more benefits. In addition, a moderator analysis was employed to examine the relationship between total training and exercise capacity and lung function (FEV1 and FEV1%). This tells us that adherence to Wuqinxi is the key to alleviating these disease-related symptoms.

Exercise capability and lung function improved following Wuqinxi training. Given that the potential mechanism of positive effects of Wuqinxi on these outcomes still remain unknown, features of Wuqinxi may partially explain the positive results. Physical movements in Wuqinxi forms involve upper-limb stretching or thoracic expansion coordinated with deep breathing. These components could be a leading contributor to stronger lung capability and diaphragm of COPD patients. For instance, Movement 1 and 3 involve simulating Tiger and Deer, respectively. COPD patients were instructed to slowly perform musculoskeletal (thoracic, diaphragmatic, abdominal) stretching, which could provide some simulations for respiratory muscles, ultimately leading to better exercise capability and lung function. It is worth emphasizing that body awareness/mental concentration may help COPD patients feel more relaxed mentally, which may reduce the number of dropouts in this special population.

Limitations of selected studies should be pointed out here so that researchers in future studies may obtain convincing findings. First, some studies lacked allocation concealment and blinded assessors, which could attenuate the interpretation of our positive results. Second, even if it is reasonable that Wuqinxi was integrated with either drug therapy or usual care in some studies, this integration not entirely able to convince us that these positive results are attributed to Wuqinxi training alone. Third, the course of disease progression varied greatly across selected studies (some studies did not report the demographic information). Thus, specific recommendations for different stages of COPD patients cannot be determined. Fourth, none of the studies employed follow-up assessment, which does not allow us to know how long the rehabilitative effects of Wuqinxi training last. Fifth, COPD patients in the selected studies were Chinese. Thus, it is not sure if study results can be generalized into other non-Chinese populations.

## 5. Conclusions

Our meta-analysis indicated that Wuqinxi, as a mind-body exercise, may have rehabilitative effects for COPD patients. With no occurrence of adverse event, clinicians could try to incorporate this training modality into their first-line rehabilitation regime for COPD patients. Given the study limitations of selected studies in this review, preliminary findings should be verified by more well-designed trial with follow-up assessment.

## Figures and Tables

**Table 1:** Summary of selected randomized controlled trials.

Reference	Location (Language)	Participant Characteristics			Intervention Program	Wuqinxi Training			Outcome Measured	Adverse Event; Follow-Up
		Sample Size (AT)	Mean Age or Age Range	Course of Disease (year)		Frequency (weekly)	Time (min)	Duration (week)		
Chen, et al. (2015) [19]	Anhui, China (Chinese)	100 (10.34%)	WQ: 58.66 (7.56) CG: 58.64(7.52)	14.52 (5.96)	WQ: Wuqinxi CG: Usual Care	4	30	24	Quality of life (CAT)	No; No
He et al. (2015) [20]	Anhui, China (Chinese)	93 (7.00%)	WQ: 58.66 (7.56) CG: 58.64 (7.52)	14.52 (5.96)	WQ: Wuqinxi + Drug Therapy CG: Drug Therapy	4	30	24	Quality of life (ZSH)	No; No
Gao et al. (2016) [21]	Guangdong, China (Chinese)	74 (2.70%)	WQ: 67.14 (9.08) CG: 66.03 (8.18)	WQ: 13.69 (5.67) CG: 14.78 (9.24)	WQ: Wuqinxi + Drug Therapy CG: Breathing training + Drug Therapy	14	30	24	Lung function (FEV1, FEV1%, FEV1/FVC) Exercise Capacity (6-MWT)	No; No
Tan et al. (2017) [22]	Guangdong, China (Chinese)	100 (0%)	WQ: 67.72 (9.26) CG: 74.24 (9.10)	NR	WQ: Wuqinxi CG: NR	7	45	24	Quality of life (CCQ)	No; No
Wei et al. (2015) [23]	Anhui, China (Chinese)	93 (7.00%)	WQ: 58.66 (7.56) CG: 58.64 (7.52)	14.52 (5.96)	WQ: Wuqinxi + Drug Therapy CG: Drug Therapy	4	30	24	Lung function (FEV1%, FEV1/FVC)	No; No
Xing et al. (2017) [24]	Tainting, China (Chinese)	93 (0%)	WQ: 57.58 (7.47) TC: 59.00 (7.84)	WQ: 7.96 (1.36) TC: 7.25 (1.58)	WQ: Wuqinxi TC: Tai chi	4	45	12	Exercise Capacity (6-MWT) Lung function (FEV1%, FEV1/FVC), Quality of life (CCQ)	No; No
Zhao et al. (2015) [25]	Zhengzhou, China (Chinese)	60 (0%)	WQ: 58.91 (5.86) CG: 56.66 (6. 43)	WQ: 6.81 (2.34) CG: 6.52 (2.42)	WQ: Wuqinxi CG: Drug Therapy	3	40	12	Lung function (FEV1, FEV1%, FEV1/FVC), Exercise Capacity (6-MWT)	No; No
Zhu et al. (2010) [26]	Nanjing, China (Chinese)	74 (5.12%)	WQ: 53.53 (10.05) WT: 55.12 (11.41) CG: 55.46 (9.87)	NR	WQ: Wuqinxi + Usual Care WT: Walking training + Usual Care CG: Usual Care	7	45	12	Lung function (FEV1, FEV1%, FEV1/FVC) Exercise Capacity (6-MWT)	No; No

Note: AT = attrition rate; WQ = Wuqinxi; CG = control group; TC = Tai chi; WT = Walking training; FEV1 = the forced expiratory volume in one second;FEV1% = percentage of the forced expiratory volume in one second; FEV1/FVC = the amount of air exhaled in the first second divided by all of the air exhaled during a maximal exhalation; 6-MWT = 6-Minute Walking Test; CCQ = Clinical Chronic Obstructive Pulmonary Disease Questionnaire; CAT = COPD Assessment Test; QLA (ZSH) = Quality of Life Assessment Form scored by Zhongshan Hospital.

**Table 2:** Study quality assessment for eligible randomized controlled trials.

Author [Reference]	Item 1	Item 2	Item 3	Item 4	Item 5	Item 6	Item 7	Item 8	Item 9	Score
Chen, et al. (2015) [19]	1	1	1	0	1	1	1	1	1	8
He, et al. (2015) [20]	1	0	1	0	1	1	1	1	0	6
Gao, et al. (2017) [21]	1	1	1	1	1	1	1	1	0	8
Tan, et al. (2016) [22]	1	0	1	0	1	1	1	1	1	7
Wei, et al. (2015) [23]	1	1	1	0	1	1	1	1	0	7
Xing, et al. (2017) [24]	1	0	1	0	1	1	1	1	1	7
Zhao, et al. (2015) [25]	1	0	1	0	1	1	1	1	1	7
Zhu, et al. (2010) [26]	1	1	1	0	1	1	1	1	0	6

Note: Item 1 = randomization; Item 2 = concealed allocation; Item 3 = similar baseline; Item 4 = blinding of assessors; Item 5 = more than 85% retention; Item 6 = missing data management (intention-to-treat analysis); Item 7 = between-group comparison; Item 8 = point measure and measures of variability; Item 9 = isolated Wuqinxi intervention; 1 = explicitly described and present in details; 0 = absent, inadequately described, or unclear.

**Table 3:** Synthesized results for the effects of Wuqinxi vs Health education.

Outcomes	Number of Comparisons	Meta-Analysis			Heterogeneity			Publication Bias
		SMD	95% CI	*p*-Value	*I*^2^ %	*Q*-Value	df (*Q*)	Egger’s Test (*e*)
6MW	5	1.18	0.530 to 1.84	0.00	84.97%	26.61	4	0.24
FEV1	4	0.44	0.12 to 0.77	0.00	33.77%	4.53	3	0.44
FEV1%	6	0.59	0.24 to 0.93	0.00	63.79%	13.81	5	0.55
FEV1/FVC	6	0.65	0.37 to 0.93	0.00	44.32%	8.90	5	0.72
CCQ	4	1.23	0.31 to 2.14	0.01	93.32%	44.92	3	0.35

Note: FEV1 = the forced expiratory volume in one second; FEV1% = percentage of the forced expiratory volume in one second; FEV1/FVC = the amount of air exhaled in the first second divided by all of the air exhaled during a maximal exhalation; 6-MWT = 6-Minute Walking Test; CCQ = Clinical Chronic Obstructive Pulmonary Disease Questionnaire.

**Table 4:** Moderator analysis for the effects of Wuqinxi vs. control intervention (continuous predictor).

Outcomes	Continuous Predictors	Number of Comparisons	*β*	95% CI	*Q*-Value	df (*Q*)	*p*-Value
6MW	Total training time	5	0.00014	0.00005 to 0.00022	10.66	1	0.001
FEV1	Total training time	4	0.00007	−0.00000 to 0.00015	3.50	1	0.06
FEV1%	Total training time	6	0.00011	0.00004 to 0.00008	9.04	1	0.003
FEV1FVC	Total training time	6	0.00008	0.00001 to 0.00015	4.38	1	0.036
CCQ	Total training time	3	0.00007	0.00000 to 0.00014	4.18	1	0.04

Note: FEV1 = the forced expiratory volume in one second; FEV1% = percentage of the forced expiratory volume in one second; FEV1/FVC = the amount of air exhaled in the first second divided by all of the air exhaled during a maximal exhalation; 6-MWT = 6-Minute Walking Test; CCQ = Clinical Chronic Obstructive Pulmonary Disease Questionnaire.

## Supplementary Materials

The following are available online at http://www.mdpi.com/1660-4601/16/1/72/s1, Table S1: The standard data for the included studies.
