# Supplementary material for: Mind-Body Exercise (Wuqinxi) for Patients with Chronic Obstructive Pulmonary Disease: A Systematic Review and Meta-Analysis of Randomized Controlled Trials
Source: Int J Environ Res Public Health. 2018 Dec 28;16(1):72. doi: 10.3390/ijerph16010072 (PMC6338907; doi:10.3390/ijerph16010072)
Supplement: Supplementary file 1 [file ijerph-16-00072-s001.pdf]

Table S1 The standard data for the included studies.

Study	Wuqinxi gourp			Control group		
	Baseline	Post-test	N	Baseline	Post-test	N
Gao, et al 2017	6WMD=332.67±23.77<br>FEV1=1.33±0.37<br>FEV1%=43.3±8.94<br>FEV1FVC=40.17±8.91	6WMD=417.56±19.73<br>FEV1=1.33±0.37<br>FEV1%=55.08±8.16<br>FEV1FVC=55.31±9.41	36	6WMD=328.67±22.86<br>FEV1=1.29±0.32<br>FEV1%=44.51±10.58<br>FEV1FVC=38.4±11.55	6WMD=350.78±23.39<br>FEV1=1.25±0.31<br>FEV1%=44.61±9.89<br>FEV1FVC=43.55±8.92	36
Xing, et al 2017	6WMD=446.92±94.10<br>FEV1%=73.58±6.35<br>FEV1FVC=57.94±5.06	6WMD=454.05±71.2<br>FEV1%=75.37±6.18<br>FEV1FVC=54.95±5.08	31	6WMD=446.78±93.52<br>FEV1%=73.25±6.31<br>FEV1FVC=61.2±4.83	6WMD=497.89±77.42<br>FEV1%=79.41±5.63<br>FEV1FVC=55.02±4.8	31
Tan et al 2016	CCQ=32.58±3.74	CCQ=17.06±3.45	50	CCQ=94.56±1.63	CCQ=34.82±4.21	50
Chen et al 2015	CCQ=20.02±4.78	CCQ=15.61±4.2	48	CCQ=20.51±4.75	CCQ=20.02±5.27	45
He et al 2015	CCQ=23.56±2.12	CCQ=21.81±1.2	48	CCQ=23.78±2.36	CCQ=23.82±2.49	45
Wei et al 2015	FEV1%=64.91±8.72<br>FEV1FVC=62.14±5.4	FEV1%=61.89±8.77<br>FEV1FVC=58.97±5.63	48	FEV1%=64.55±6.03<br>FEV1FVC=61.26±6.25	FEV1%=58.82±5.76<br>FEV1FVC=54.31±6.45	45
Zhao, et al 2015	6WMD=398.96±21.41<br>FEV1=85.8±4.12<br>FEV1%=55.68±4.69<br>FEV1FVC=64.34±3.96	6WMD=439.04±31.27<br>FEV1=86±3.71<br>FEV1%=55.92±3.95<br>FEV1FVC=64.62±3.65	30	6WMD=403.12±20.97<br>FEV1=87.65±4.94<br>FEV1%=57.46±4.31<br>FEV1FVC=65.44±3.36	6WMD=401.59±26.67<br>FEV1=86.65±4.29<br>FEV1%=57.77±3.89<br>FEV1FVC=65.17±2.82	30
Zhu, et al 2010 (1)	6WMD=342.3±48.7<br>FEV1=1.48±0.29<br>FEV1%=55.02±10.87<br>FEV1FVC=64.92±10.74	6WMD=447.6±61.7<br>FEV1=1.74±0.82<br>FEV1%=67.07±15.98<br>FEV1FVC=76.14±10.67	26	6WMD=334.9±38.6<br>FEV1=1.45±0.3<br>FEV1%=54.09±9.62<br>FEV1FVC=64.15±8.76	6WMD=456.9±43.4<br>FEV1=1.63±0.46<br>FEV1%=60.67±14.61<br>FEV1FVC=70.97±12.87	27
Zhu, et al 2010 (2)	6WMD=342.3±48.7<br>FEV1=1.48±0.29<br>FEV1%=55.02±10.87<br>FEV1FVC=64.92±10.74	6WMD=447.6±61.7<br>FEV1=1.74±0.82<br>FEV1%=67.07±15.98<br>FEV1FVC=76.14±10.67	26	6WMD=324.6±52.82<br>FEV1=1.42±0.45<br>FEV1%=53.07±12.02<br>FEV1%=63.51±9.58	6WMD=319.5±41.4<br>FEV1=1.41±0.18<br>FEV1%=52.56±10.73<br>FEV1%=62.76±10.04	21
